# Pharmacological Properties of DOV 315,090, an ocinaplon metabolite

**DOI:** 10.1186/1471-2210-8-11

**Published:** 2008-06-13

**Authors:** Dmytro Berezhnoy, Maria C Gravielle, Scott Downing, Emmanuel Kostakis, Anthony S Basile, Phil Skolnick, Terrell T Gibbs, David H Farb

**Affiliations:** 1 Laboratory of Molecular Neurobiology, Department of Pharmacology & Experimental Therapeutics, Boston University School of Medicine, 715 Albany St., Boston, MA 02118, USA; 2 DOV Pharmaceutical, Inc, 150 Pierce St., Somerset, NJ 08873-4185, USA

## Abstract

**Background:**

Compounds targeting the benzodiazepine binding site of the GABA_A_-R are widely prescribed for the treatment of anxiety disorders, epilepsy, and insomnia as well as for pre-anesthetic sedation and muscle relaxation. It has been hypothesized that these various pharmacological effects are mediated by different GABA_A_-R subtypes. If this hypothesis is correct, then it may be possible to develop compounds targeting particular GABA_A_-R subtypes as, for example, selective anxiolytics with a diminished side effect profile. The pyrazolo[1,5-a]-pyrimidine ocinaplon is anxioselective in both preclinical studies and in patients with generalized anxiety disorder, but does not exhibit the selectivity between α_1_/α_2_-containing receptors for an anxioselective that is predicted by studies using transgenic mice.

**Results:**

We hypothesized that the pharmacological properties of ocinaplon *in vivo *might be influenced by an active biotransformation product with greater selectivity for the α_2 _subunit relative to α_1_. One hour after administration of ocinaplon, the plasma concentration of its primary biotransformation product, DOV 315,090, is 38% of the parent compound. The pharmacological properties of DOV 315,090 were assessed using radioligand binding studies and two-electrode voltage clamp electrophysiology. We report that DOV 315,090 possesses modulatory activity at GABA_A_-Rs, but that its selectivity profile is similar to that of ocinaplon.

**Conclusion:**

These findings imply that DOV 315,090 could contribute to the action of ocinaplon in vivo, but that the anxioselective properties of ocinaplon cannot be readily explained by a subtype selective effect/action of DOV 315,090. Further inquiry is required to identify the extent to which different subtypes are involved in the anxiolytic and other pharmacological effects of GABA_A_-R modulators.

## Background

GABA_A _receptors (GABA_A_-R) are pentameric membrane proteins that belong to the superfamily of cys-loop ligand-gated ion channels (LGIC), which operate as GABA-gated Cl^-^-selective channels. GABA_A_-R mediate most of the fast inhibitory neurotransmission in the CNS [1-3]. Initially, two subunits of the GABA_A_-R named α and β were purified [4,5] and subsequently their cDNAs were isolated [6]. Twenty related GABA_A_-R subunits have been so far identified in mammals (α_1–6_, β_1–4_, γ_1–3_, δ, ε, π, θ, and ρ_1–3 _[7,8]), yielding a high degree of potential diversity. If all of these subunits could randomly co-assemble, more than one hundred thousand GABA_A_-R subtypes with distinct subunit composition and arrangement would be formed [9]. The composition of the most abundant GABA_A_-R type in the CNS is αβγ, and immunohistochemistry studies suggest that receptors containing α_1_, β_2/3 _and γ_2 _subunits are the most widespread GABA_A_-R subtype in adult mammalian brain and represent about 50% of the total receptor pool [2,10].

Typical αβγ GABA_A_-Rs harbor two agonist (GABA) binding sites located at the two α/β subunit interfaces [2,11]. The function of GABA_A_-Rs can be modulated by various compounds acting at different allosteric sites located on GABA_A_-Rs. The benzodiazepine (BZD) site, which is located at an α/γ interface [12,13], is the most frequently targeted site for therapeutic agents, and ligands that enhance GABA_A_-R function through positive modulation at this site possess anxiolytic, sedative, myorelaxant, anesthetic and amnestic properties [2,3,10,14]. Based on pharmacological studies in transgenic mice, it has been proposed that GABA_A_-Rs can be classified into the following pharmacological classes according to the effects of BZ site ligands: α_1_-containing receptors (GABA_A1_) that mediate sedative effects; α_2_-containing receptors (GABA_A2_) that mediate anxiolytic effects; α_3_-containing receptors (GABA_A3_) that mediate myorelaxation; and α_5_-containing receptors (GABA_A5_) that are involved in learning and memory processes [7,15,16]. This classification is consistent with the sedative/hypnotic profile of GABA_A1_-preferring compounds such as zolpidem and zaleplon [17], but pharmacological studies in wild-type animals and in man have raised questions regarding the attribution of anxiolytic effects to GABA_A2 _receptors. In particular, a number of compounds have been identified that exhibit an anxioselective profile *in vivo *despite lacking the expected GABA_A2 _selectivity. A series of compounds with mixed preference for α_2_/α_3_-containing receptors has been reported to produce robust anxiolysis in animals without noticeable sedation, including one compound that exhibits selectivity for α_3_-containing receptors [18-21]. Other compounds, such as ocinaplon [22] and DOV 51,892 [23], are anxiolytic in humans and animals without undesired side effects such as sedation and myorelaxation, but do not exhibit strong selectivity among GABA_A_-Rs sensitive to benzodiazepines (that is, those receptors containing α_1–3 _and/or α_5_-subunits)

**Figure 1:**
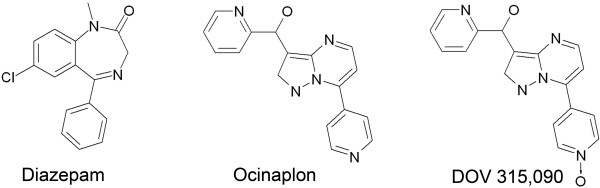
Structures of diazepam, ocinaplon and DOV 315,090.

One hypothesis that could explain the anxioselective profile of ocinaplon is the presence of one or more biotransformation products that exhibit selectivity at GABA_A2 _receptors. To test this hypothesis, we characterized the pharmacological properties of the major biotransformation product of ocinaplon in dogs, rats and man, DOV 315,090 (Fig. 1), using *in vitro *radioligand binding and two-electrode voltage-clamp electrophysiology. We now report that like its parent compound, DOV 315,090 acts as a positive modulator at GABA receptors, and like its parent, does not exhibit marked selectivity among α_1–3 _and α_5 _containing receptors. Thus, while DOV 315,090 may contribute to the pharmacological actions of ocinaplon, the anxioselective profile of ocinaplon cannot be explained on the basis of enhanced subunit selectivity on the part of DOV 315,090.

## Methods

### Radioligand Binding Assays

HEK293 cells (CRL 1573, American type Culture Collection, Rockville, MD, USA) were cultured in Dulbecco's modified Eagle's medium (D-MEM, Invitrogen, Carlsbad, CA, USA) supplemented with 10% fetal bovine serum (Invitrogen, Carlsbad, CA, USA) and 1% MEM Non-Essential Amino Acids Solution (Invitrogen, Carlsbad, CA, USA). cDNAs encoding rat GABA_A_-R subunits were in the following vectors: α_1 _and α_5 _in pRc/CMV, α_2_, α_3_, γ_2S _and γ_3 _in pcDNA3 and β_2 _in pcDNA1. The cells were transiently transfected (5 μg of each cDNA per 100 mm dish) using FuGene™ (Roche Diagnostics Corporation) at a 3:1 FuGene:DNA ratio. Transfection efficiency was 50–80% as measured by co-transfection with green fluorescent protein cDNA (2.5 μg/100 mm dish). Forty-eight hours after transfection, cells were washed with ice-cold PBS, harvested and homogenized. Cell homogenates were centrifuged (100,000 *g*, 25 min) and washed three times by homogenization in ice-cold PBS buffer followed by centrifugation at 100,000 *g *for 25 min. The final pellets were stored at -20°C until needed.

For competition binding, 100 μg of membrane protein was incubated in 500 μl of PBS buffer with 0.5 nM [^3^H]Ro15–1788 (78.6 Ci/mmol, PerkinElmer Life Sciences) in the presence of diazepam (1 nM – 10 μM, Sigma-Aldrich), ocinaplon (0.1 – 250 μM, DOV Pharmaceuticals) or DOV 315,090 (0.1 – 50 μM, DOV Pharmaceuticals) for 1 h at 0°C. The samples were then diluted with 5 ml of ice-cold buffer and filtered under vacuum through glass-fiber filters (GF/B Whatman). Filters were washed 3 times with 5 ml of buffer and the radioactivity was quantitated by liquid scintillation counting in 5 ml of Ecolite scintillation fluid (ICN). Non-specific binding determined in the presence of 100 μM Ro 15–1788 (Sigma-Aldrich) was subtracted from total binding to calculate specific binding. Data were analyzed by non-linear regression (Prism, Graph-Pad software).

### Recording of GABA-Gated Currents from GABA_A _Receptors Expressed in Xenopus Oocytes

cRNAs encoding GABA_A_-R α_1_, α_2_, α_3_, α_5_, β_2 _and γ_2S _subunits were injected into oocytes from *Xenopus laevis*. Forty-eight hours later, measurements of the effects of diazepam, ocinaplon and DOV 315,090 on GABA-gated Cl^- ^currents from oocytes expressing GABA_A_-Rs were performed using a Warner TEVC amplifier (Warner Instruments, Inc., Foster City, CA) (Park-Chung et al., 1999). GABA (Sigma) was prepared as a 1 M stock solution in ND96. Microelectrodes of 1–3 MΩ when filled with 3 M KCl were used to record from oocytes in a recording chamber continuously perfused with ND-96 buffer solution. During data acquisition, oocytes were clamped at a holding potential of -70 mV. Drugs were applied by perfusion at a rate of approximately 50 μl sec^-1 ^for 20 s followed by a 120 s wash. At the end of each experiment 3 μM of diazepam was applied as a potentiation control. All experiments were performed at room temperature (22–24°C).

GABA concentration-response data was obtained for each subunit combination, and the GABA EC_10 _was determined by nonlinear regression using the logistic equation. This concentration of GABA was used for modulation studies. Peak current measurements were normalized and expressed as a fraction of the peak control current measurements. Control responses to an EC_10 _concentration of GABA were re-determined after every 2 – 4 applications of modulator + GABA. Percent potentiation is defined as [I_(GABA + Drug)_/I_GABA_)-1] × 100, where I_(GABA + Drug) _is the current response in the presence of diazepam, and I_GABA _is the control GABA current. Potentiation data from each oocyte was fitted to the equation Potentiation = E_max _× [Drug]/([Drug + EC_50_) by non-linear regression (Prism, Graph-Pad software). Due to a decline in the response at high diazepam concentrations, concentrations of diazepam above 3 μM were excluded from the fit. Some oocytes expressing α1β1γ2 receptors appeared to exhibit a biphasic modulatory response to diazepam, suggesting the possible presence of an additional component of modulation with a sub-nM EC_50_. For 6 of 8 oocytes, the fit was significantly improved by adding a second, higher-potency component of modulation, but the affinity of this second component was not well resolved in fitting due to its small amplitude. Given the lack of consistency of this possible high affinity effect, we have omitted it in fitting our concentration-effect curves. The choice of fitting to a monophasic or biphasic equation had only a small effect on the EC_50 _for the major component of modulation. For diazepam, the mean EC_50 _of the major component was increased from 35 nM to 42 nM when a two-component fit was used for those oocytes in which it produced a significant improvement in the sum of squares.

## Results

### Biotransformation of ocinaplon into DOV 315,090 *in vivo*

**Figure 2:**
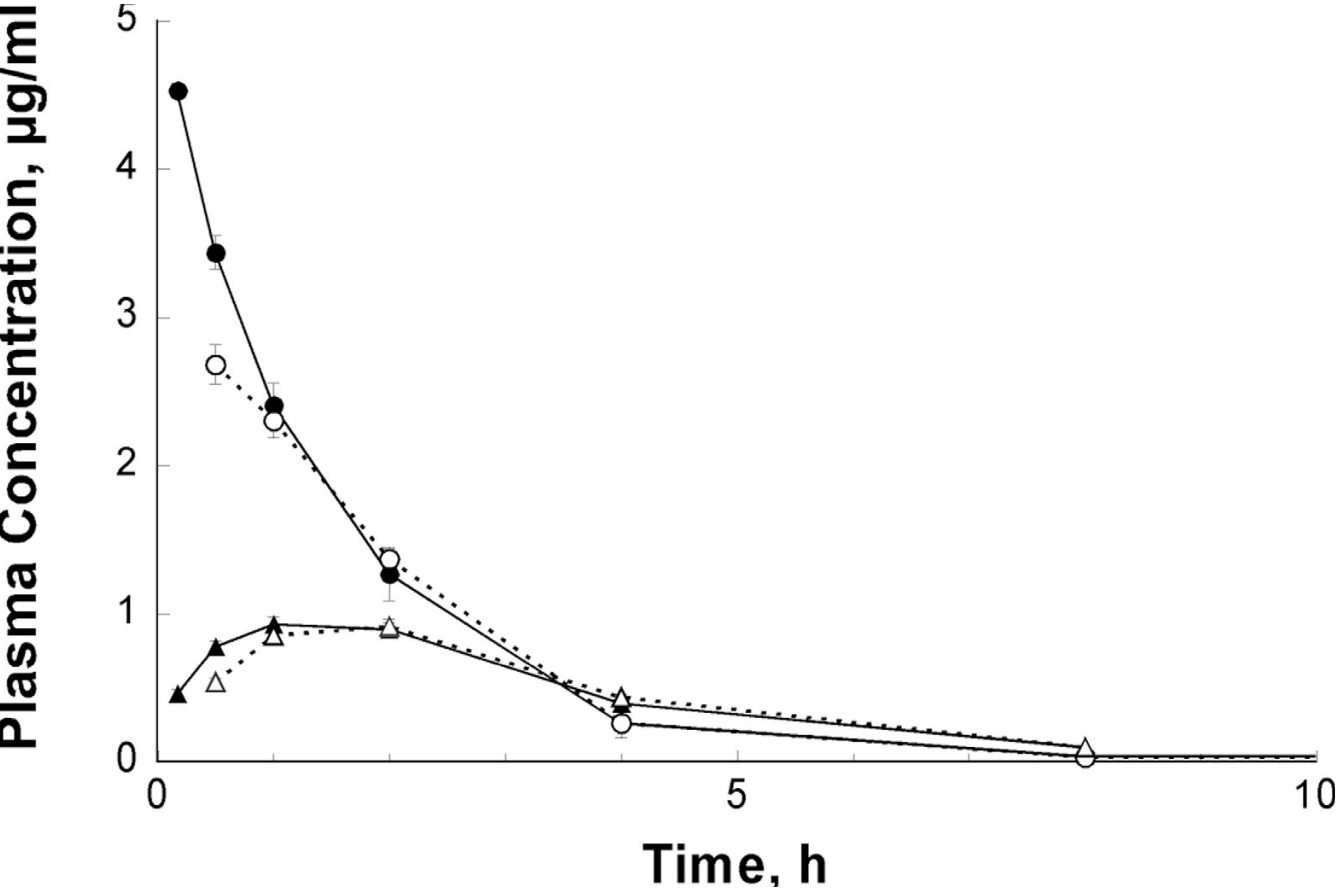
**Pharmacokinetics of ocinaplon and DOV 315,090.** Blood levels of ocinaplon (●,○) and DOV 315090 (▲,△) were determined at various times after i.v. (●,▲) or oral (○,△) administration of 5 mg/kg ocinaplon to rats. Plotted results do not include one animal that exhibited a low blood level (0.47 μg/ml) of ocinaplon at the initial 10 min time point after oral administration and proportionally lower levels of both compounds throughout the duration of the experiment. This animal may have regurgitated a portion of the dose (of the suspension).

As shown in Figure 2, DOV 315,090 appears rapidly in plasma following i.v. or oral administration of a behaviorally active dose of ocinaplon (5 mg/kg) to rats. At 1 h, corresponding to the time at which the anticonflict effect of ocinaplon was evaluated [22], the plasma concentration of DOV 315,090 is ~38% of the concentration of parent compound.

### Comparison of the binding properties of diazepam, ocinaplon and DOV 315,090

**Figure 3:**
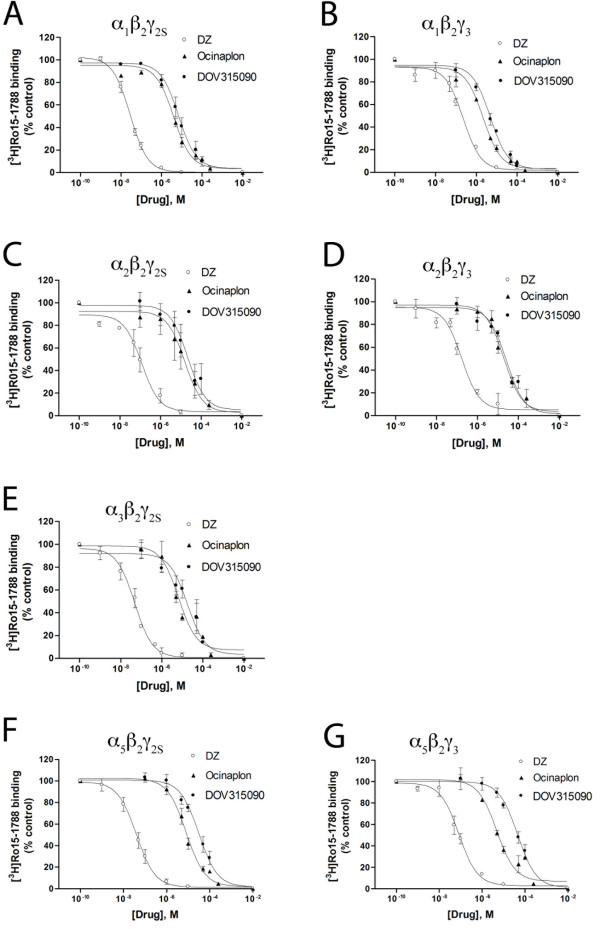
**Displacement curves of [^3^H]Ro 15–1788 binding by diazepam (DZ), ocinaplon and DOV 315,090 in homogenates of HEK293 cells transfected with different subunit combinations.** Smooth curves are calculated from the mean parameter values in Table 1.

Figure 3 and Table 1 document the binding properties of diazepam, ocinaplon and DOV 315,090 in HEK293 cells expressing different GABA_A_-R subunit combinations. Examination of binding constants shows that ocinaplon and DOV 315,090 have lower affinity than diazepam at all of the receptor subunit combinations tested. The binding profile of DOV 315,090 is similar to that of ocinaplon, with little selectivity among the subunit combinations tested. In contrast to diazepam, which exhibits markedly lower affinity for α_1_β_2_γ_3 _and α_2_β_2_γ_3 _receptors than for α_1_β_2_γ_2 _s and α_2_β_2_γ_2 _s receptors, replacement of γ_2S _with γ_3 _had little effect on the affinity of either ocinaplon or DOV 315,090 for any subunit combination (Table 1). Also, whereas diazepam has similar affinity for α_1_-containing and α_2_-containing receptors, both ocinaplon and DOV 315,090 have 3–4 fold lower affinity for α_2_-containing receptors. Specific [^3^H]Ro15–1788 or [^3^H]flunitrazepam binding to membrane preparations from cells transfected with α_3_, β_2 _and γ_3 _subunits was not detected, suggesting that these subunits failed to assemble in the HEK293 cells.

**Table 1:** Binding affinity of diazepam, ocinaplon and DOV 315,090 for GABA_A_-Rs with different subunit composition.

Receptor Type		**α_1_β_2_γ_2S_**	**α_1_β_2_γ_3_**	**α_2_β_2_γ_2S_**	**α_2_β_2_γ_3_**	**α_3_β_2_γ_2S_**	**α_5_β_2_γ_2S_**	**α_5_β_2_γ_3_**
diazepam (DZ)	IC_50 _(μM)	0.03	0.22	0.04	0.21	0.05	0.03	0.09
	pIC_50_	7.54 ± 0.09	6.67 ± 0.08	7.50 ± 0.10	6.80 ± 0.26	7.32 ± 0.08	7.57 ± 0.13	7.09 ± 0.13

ocinaplon (OC)	IC_50 _(μM)	6.3	2.3	24	20	7.7	9.6	10
	pIC_50_	5.20 ± 0.14	5.65 ± 0.01	4.62 ± 0.14	4.74 ± 0.15	5.12 ± 0.06	5.02 ± 0.03	5.01 ± 0.18
	IC_50_/DZ IC_50_	218	10.5	759	115	158	355	120

DOV 315,090	IC_50 _(μM)	7.0	5.5	24	20	9.3	22	27
	pIC_50_	5.19 ± 0.12	5.27 ± 0.07	4.63 ± 0.05	4.72 ± 0.09	5.08 ± 0.14	4.67 ± 0.08	4.58 ± 0.09
	IC_50_/DZ IC_50_	220	25	760	120	170	790	323
	IC_50_/OC IC_50_	1.02	2.40	0.89	0.98	1.09	2.24	2.59

IC_50 _values were calculated from [^3^H]Ro15–1788 displacement curves using non-linear regression analysis for each independent experiment. pIC_50 _values are averages of the negative logarithms of IC_50_s. Results from each experiment (n = 3) were fitted independently and fitted parameters were averaged to calculate means and SEM. EC_50 _values were averaged as their negative logarithms (pIC_50_).

### Modulation of GABA_A_-R function by diazepam, ocinaplon and DOV 315,090

Consistent with previous studies [22,23], the potency and efficacy of ocinaplon were lower than diazepam at the four receptor subtypes analyzed. The highest efficacy was observed at receptors containing α_3 _subunits (Table 2). DOV 315,090 also exhibited the highest maximal potentiation at α_3_-containing receptors; however, its E_max _values were similar to those of diazepam at receptors containing α_1 _or α_3 _subunits (Table 2).

**Table 2:** Properties of diazepam, ocinaplon and DOV315090 determined by two-electrode voltage clamp electrophysiology using Xenopus oocytes injected with cRNA.

Receptor Type		**α_1_β_2_γ_2S_**	**α_2_β_2_γ_2S_**	**α_3_β_2_γ_2S_**	**α_5_β_2_γ_2S_**
diazepam (DZ)	EC_50 _(μM)	0.04 (8)	0.03 (10)	0.092 (5)	0.025 (5)
	pEC_50_	7.46 ± 0.07	7.60 ± 0.044	7.04 ± 0.05	7.51 ± 0.11
	E_max_, %	144 ± 8.0	157 ± 14	232 ± 31	224 ± 24

ocinaplon (OC)	EC_50 _(μM)	2.93 (4)	9.12 (5)	8.01 (4)	3.5 (4)
	pEC_50_	5.57 ± 0.11	5.04 ± 0.03	5.16 ± 0.14	5.48 ± 0.07
	EC_50_/DZ EC_50_	77	350	87	139
	E_max_, %	132 ± 8	150 ± 6	181 ± 18	84 ± 4
	E_max_/DZ E_max_	0.91	0.95	0.78	0.37

DOV315090 (MET)	EC_50 _(μM)	4.87 (4)	12.5 (4)	10.21 (4)	10.14 (4)
	pEC_50_	6.32 ± 0.05	4.92 ± 0.09	5.00 ± 0.05	5.03 ± 0.10
	EC_50_/DZ EC_50_	128	482	111	405
	EC_50_/OC EC_50_	1.66	1.37	1.27	2.92
	E_max_, %	192 ± 4	139 ± 23 *	340 ± 35 *	68 ± 8
	E_max_/DZ E_max_	1.33	0.88	1.46	0.30
	E_max_/OC E_max_	1.45	0.92	1.87	0.81

Drugs were prepared from DMSO stock solution prior to experiment, EC_10_s of GABA were used, errors are SEM of fitted parameter values from the number of oocytes given in parentheses. Results from each oocyte were fitted independently and fitted parameters were averaged to calculate means and SEM. EC_50 _values were averaged as their negative logarithms (pEC_50_) * For these two cases, the extrapolated E_max _exceeded the observed maximum observed potentiation by over 25%, but parameter SEM was not substantially increased, indicating that range of concentrations was adequate to project E_max_. Higher drug concentrations could not be used due to solubility constraints.

**Figure 4:**
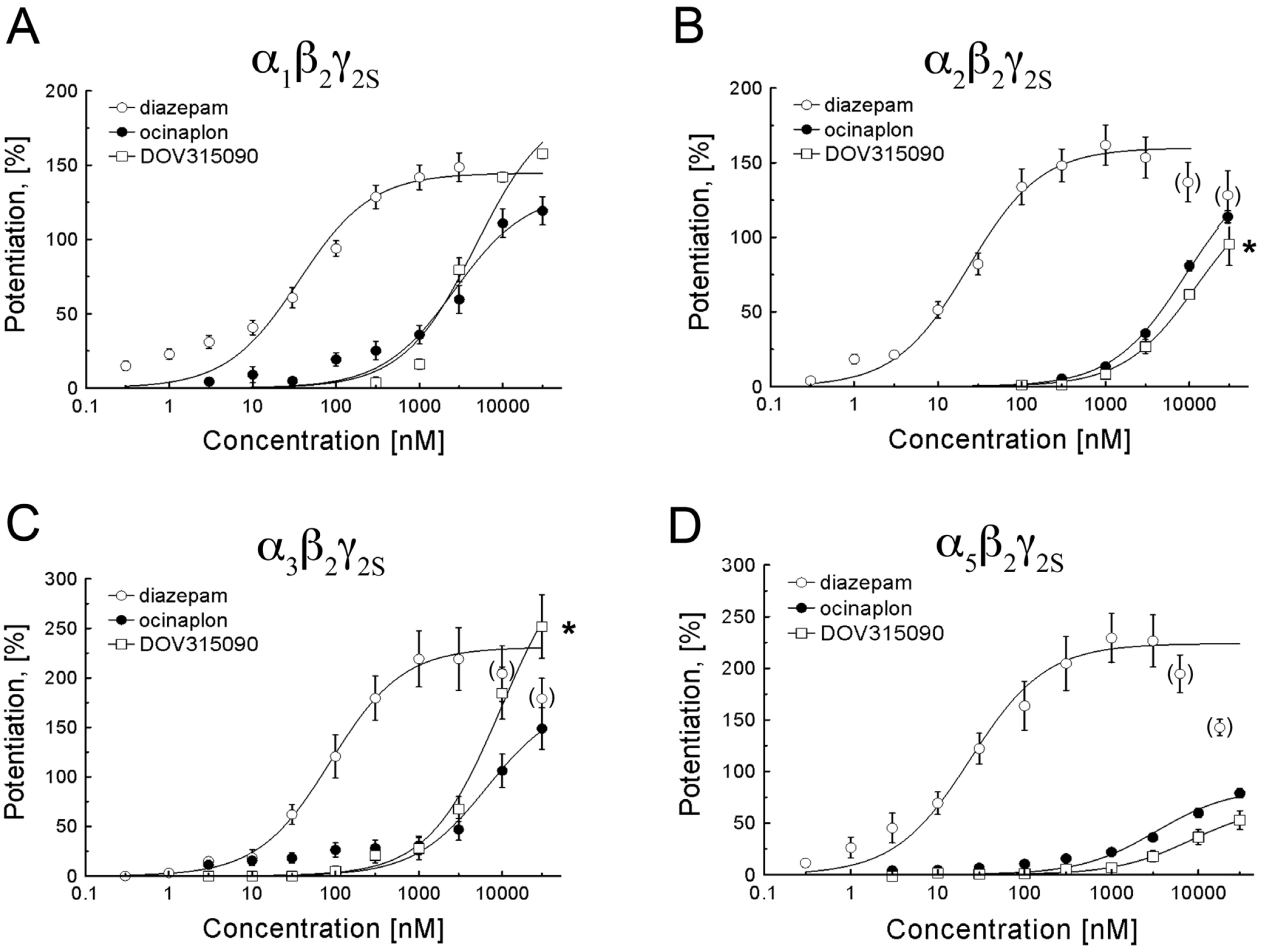
**Potentiation of GABA-gated currents by diazepam, ocinaplon and DOV 315,090.** Rat GABA_A_-Rs consisting of α_1_β_2_γ_2S_, α_2_β_2_γ_2S_, α_3_β_2_γ_2S _and α_5_β_2_γ_2S _subunits were expressed in *Xenopus *oocytes. Potentiation was determined using an EC_10 _concentration of GABA (~10 μM for α_1_β_2_γ_2S_, α_2_β_2_γ_2S _and α_3_β_2_γ_2S_; ~5 μM for the α_5_β_2_γ_2S_). Curves were calculated by normalizing values of relative currents obtained following administration of diazepam (○), ocinaplon (●) or DOV 315,090 (□) in the presence of GABA (from at least four oocytes harvested from at least two batches) to the value obtained following application of GABA. The dose-response curves of diazepam were fitted up to 3 μM. Higher concentrations (in parentheses) were excluded from the fit due to a decline in potentiation at higher concentrations. Smooth curves are calculated based on mean parameter values given in Table 2. Asterisks indicate fits for which the extrapolated E_max _is more than 25% greater than the maximum potentiation observed at highest drug concentration.

DOV 315,090 and ocinaplon exhibited similar efficacies (150% vs. 139% potentiation, respectively) and EC_50_s (12.5 μM vs. 9.12 μM, respectively, n = 4) at α_2_β_2_γ_2S _receptors (Figure 4, Table 2). In contrast, whereas ocinaplon and DOV 315,090 were approximately equipotent at α_3_β_2_γ_2S _receptors (EC_50 _= 8.01 μM and 10.21 μM, respectively), the efficacy of DOV 315,090 was almost 1.87 fold greater than that of ocinaplon (340% vs 181% potentiation) (Figure 4, Table 2). Finally, DOV 315,090 was less efficacious and potent than ocinaplon at α_5_β_2_γ_2S _receptors (Figure 4, Table 2). The rank order of potency (EC_50_) of the pyrazolopyrimidines at enhancing GABA-gated chloride currents in receptors containing different α subunits was: α_2_≈α_3_≈α_5 _< α_1 _for DOV 315,090, compared to α_2_≈α_3 _< α_5_≈α_1 _for ocinaplon. Furthermore, DOV 315,090 and ocinaplon had different efficacy (E_max_) profiles; the rank order of absolute efficacy was α_5 _< α_2 _< α_1 _< α_3 _for DOV 315,090, as compared with α_5 _< α_1 _< α_2 _< α_3 _for ocinaplon.

## Discussion

In the CNS, classical 1,4-BZDs such as diazepam, as well as other ligands of the BZD binding site, act on GABA_A_-Rs that are composed of α, β, and γ subunits. The majority of GABA_A _receptors contain α_1–6_, β_2/3 _and γ_2 _subunits, whereas the β_1 _and γ_1/3 _subunits have very restricted patterns of expression [2]. It has been shown that BZD pharmacology is primarily dependent upon the α subunit subtype present (α_1–3 _or α_5_), whereas receptors containing α_4 _or α_6 _subunits are insensitive to "classical" 1,4-BZDs [7,24,25]. Studies of animals in which genes coding for specific α subunits have been deleted or mutated to eliminate BZD sensitivity (e.g. the α_1_H101R mutation, which disrupts the BZD binding site) led to the hypothesis that the sedative effects of the BZDs are mediated by α_1_-subunit containing receptors (designated GABA_A1_-R), whereas anxiolytic effects are mediated by α_2_-subunit containing receptors (GABA_A2_-R) [7,17,26,27]. GABA_A_-Rs containing α_5 _subunits are thought to be responsible for the impairment of learning and memory that is induced by BZDs [28]. These finding raised the attractive prospect that BZD-like drugs that specifically target GABA_A_-Rs that contain a specific α-subunit will be able to produce the intended pharmacological effect (e.g sedation or anxiolysis) with reduced incidence of side effects. Because BZD-like drugs function as allosteric modulators and do not occupy the GABA binding site, specificity may be potentially achieved on the basis of either differences in potency or on differences in modulatory efficacy at specific receptor subtypes.

Compounds such as zolpidem and zaleplon, which exhibit higher affinity for α1-containing receptors relative to other subtypes, have been promoted as sedative agents, driven in part by the hypothesis that selectivity for GABA_A1_-Rs would translate into an improved side-effect profile, particularly with respect to tolerance, withdrawal, and abuse liability. Although these compounds are effective sedative agents, consistent with the identification of GABA_A1_-Rs as mediating sedation, the selectivity of these compounds for GABA_A1_-Rs vs. GABA_A_-Rs containing other α-subunits is generally an order of magnitude or less, and it is unclear to what extent the hypothesized benefits are achieved in clinical practice [17].

However, the situation is less clear for compounds possessing anxiolytic properties. Recently published articles describe the pharmacological properties of two novel anxioselective compounds – ocinaplon [22] and DOV 51892 [23]. These compounds do not exhibit a marked selectivity among GABA_A_-Rs containing different diazepam-sensitive subunits (e.g. α_1–3 _and α_5_), yet are reported to be anxioselective, lacking sedative and myorelaxant side effects at anxiolytic doses. In particular, DOV 51892 exhibits higher efficacy than diazepam at GABA_A1_-Rs.

The classic BZD diazepam has been shown to act with high efficacy and similar potency across a broad spectrum of GABA_A_-Rs [1,10,22] (Table 2). This lack of selectivity with respect to either potency or efficacy among the major GABA_A_-R types have been hypothesized to account for the side effects associated with the use of diazepam when used as an anxiolytic, which include sedation, myorelaxation, narcosis, and amnesia. However, as has been confirmed by *in vivo *behavioral studies, such side effects are not observed with ocinaplon (e.g. in motor activity test, inclined screen and rod walking) or for DOV 51892 (e.g. rotarod and grip strength tests), even at doses well in excess of those that enhanced punished responding in the thirsty rat test [22,23]. Further, ocinaplon is an effective anxiolytic in humans at doses that do not produce BZD-like side effects [22]. The present study was designed to test whether the anxioselective profile of ocinaplon is due to metabolism into subtype-selective metabolites. Our pharmacokinetic data demonstrate that in rats, the major metabolite of ocinaplon is a 4'-N' oxide, DOV 315,090. Whereas DOV 315,090 is active as a GABA_A_-R modulator, and its *in vitro *binding affinities for recombinant α_1_β_2_γ_2S_, α_2_β_2_γ_2S_, and α_3_β_2_γ_2S _receptors differ only marginally from ocinaplon, its affinity for α_5_β_2_γ_2S _receptors is only slightly lower than that of ocinaplon (~2-fold).

Comparison of the pharmacological profile of ocinaplon and DOV 315,090 using two electrode voltage clamp electrophysiology (Table 2) shows that the greatest difference in efficacy occurred at α_3_β_2_γ_2S _receptors. Although a clear maximum was not attained due to solubility limits, the extrapolated maximum potentiation by DOV 315,090 was 1.87-fold greater, followed by a 1.45-fold difference at α_1_β_2_γ_2S _receptors compared to ocinaplon. In contrast, maximum potentiation by DOV 315,090 was lower than that of ocinaplon at the α_5_β_2_γ_2S _receptor subtype. The efficacies of DOV 315,090 and ocinaplon at α_2_β_2_γ_2S _receptors were similar.

These results do not support the hypothesis that the anxioselective profile of ocinaplon is attributable to enhanced selectivity of its metabolite DOV 315,090 for α_2_-containing receptors. Thus, compared to ocinaplon, DOV 315,090 does not exhibit enhanced affinity or potency for α_2_-containing receptors over α_1_-containing receptors, whereas the difference in efficacy favors α_3_-, α_5_-, or α_1_-containing receptors over α_2_-containing receptors. The present experiments examined GABA_A_-Rs in two different heterologous expression systems (*Xenopus *oocytes and HEK 293 cells), which may be lacking modulatory proteins or regulatory mechanisms that are only present in neurons. While we cannot exclude the possibility that such interactions somehow confer differences in modulator binding or efficacy, such a hypothesis would require that such interactions modify the structure of the benzodiazepine binding site, which is located in the extracellular domain of the GABA_A_-R, in such a way as to selectively alter its interactions with different ligands.

Recent studies suggest that GABA_A3_-Rs receptors are also important in mediating anxiolysis [18,20,31-34]. DOV 315,090 has relatively high efficacy at α_3_β_2_γ_2S_, so it is likely that modulation of GABA_A3_-Rs by DOV 315,090 contributes to the anxioselective profile of ocinaplon; however, adipiplon (NG2-73), an α_3_-selective positive modulator, has been reported to have sedative/hypnotic activity [35], suggesting that α_3 _selectivity is not sufficient to confer anxioselectivity.

In summary, transgenic mice in which the BZD recognition site of the α_2 _subunit is disabled exhibit reduced diazepam sensitivity in behavioral tests considered to be predictive of anxiolytic activity, and a similar modification to the α_1 _subunit reduces sensitivity in tests held to be predictive of sedation [15,26]. These observations have led to optimism that it will be possible to achieve the long-desired goal of developing a nonsedating anxiolytic [36]. And indeed, there has been substantial progress in identifying such compounds [19-22,31,37-40], yet ironically, they do not in general conform to the expected paradigm of favoring α_2_-containing over α_1_-containing receptors. This suggests that anxiolysis in humans may prove to be more complex than is suggested by a simple reading of the results from transgenic mice in behavioral models thought to be indicative of anxiety. It remains to be determined whether differences in the design of the behavioral assays [41,42], interspecies differences [43,44], or a combination of these factors account for these discrepancies. Translating such promising results into clinically useful compounds is likely to require an improved understanding of the ways in which BZD-like ligands act at different GABA_A_-R subtypes and the consequences of these effects upon neural system-mediated behavioral outputs.

## Conclusion

1. DOV315090 is a major metabolite of the anxioselective GABA_A_-R modulator ocinaplon.

2. DOV 315,090 possesses modulatory activity at α_1_-, α_2_-, α_3_-, and α_5_-containing GABA_A_-Rs with a selectivity profile similar to that of ocinaplon.

3. The anxioselective properties of ocinaplon, demonstrated in both preclinical and clinical studies, are not a consequence of enhanced subtype selectivity by DOV315090.

## Abbreviations

cDNA: complementary deoxyribonucleic acid; cRNA: complementary ribonucleic acid; DOV 51892: (7-(2-chloropyridin-4-yl)pyrazolo- [1,5-*a*]-pyrimidin-3-yl](pyridin-2-yl)methanone); ocinaplon, (2-pyridinyl [7-(4-pyridinyl)pyrazolo[1,5-a]pyrimidin-3-yl]methanone); DOV 315,090: (7-(1-Oxidopyridin-1-ium-4-yl)pyrazolo [1,5-a]pyrimidin-3-yl)(pyridin-2-yl)methanone, GABA, γ-aminobutyric acid; I_GABA_: GABA-gated current.

## Authors' contributions

DB carried out electrophysiological recordings. MCG carried out radioligand binding experiments. EK performed initial electrophysiological experiments. SD developed the data-acquisition hardware and software used in this study. TTG participated in the design of the study, performed the statistical analysis and participated in manuscript preparation. DHF participated in the design of the study and participated in manuscript preparation. PS directed development of ocinaplon at DOV Pharmaceuticals and participated in manuscript preparation. ASB identified major ocinaplon metabolite and participated in manuscript preparation. All authors read and approved the final manuscript.
